# Targeting myeloid-derived suppressor cells in tumor immunotherapy: Current, future and beyond

**DOI:** 10.3389/fimmu.2023.1157537

**Published:** 2023-03-17

**Authors:** Yang Zhao, Junfeng Du, Xiaofei Shen

**Affiliations:** ^1^ State Key Laboratory of Membrane Biology, Institute of Zoology, Chinese Academy of Sciences, Beijing, China; ^2^ Beijing Institute for Stem Cell and Regenerative Medicine, Beijing, China; ^3^ Department of General Surgery, The 7th Medical Center, Chinese People’s Liberation Army General Hospital, Beijing, China; ^4^ Department of General Surgery, Affiliated Drum Tower Hospital of Nanjing University Medical School, Nanjing, China

**Keywords:** myeloid-derived suppressor cells, tumor immunotherapy, tumor immune microenvironment, combinatorial strategies, cell therapy

## Abstract

Myeloid-derived suppressor cells (MDSCs) are one of the major negative regulators in tumor microenvironment (TME) due to their potent immunosuppressive capacity. MDSCs are the products of myeloid progenitor abnormal differentiation in bone marrow, which inhibits the immune response mediated by T cells, natural killer cells and dendritic cells; promotes the generation of regulatory T cells and tumor-associated macrophages; drives the immune escape; and finally leads to tumor progression and metastasis. In this review, we highlight key features of MDSCs biology in TME that are being explored as potential targets for tumor immunotherapy. We discuss the therapies and approaches that aim to reprogram TME from immunosuppressive to immunostimulatory circumstance, which prevents MDSC immunosuppression activity; promotes MDSC differentiation; and impacts MDSC recruitment and abundance in tumor site. We also summarize current advances in the identification of rational combinatorial strategies to improve clinical efficacy and outcomes of cancer patients, *via* deeply understanding and pursuing the mechanisms and characterization of MDSCs generation and suppression in TME.

## 1 Introduction

Accumulating evidence shows that the formation of the tumor immune microenvironment (TIME) is closely related to tumor malignant development and metastasis. The occurrence and progression of tumors is a complex pathophysiological process. Most researchers believe that the TIME includes: 1) secretion of immunosuppressive factors, including interleukins, chemokines, growth factors, and other cytokines, inducing inflammatory responses and forming a local milieu conductive to tumor propagation (1, 2); and 2) many immune cell components, such as myeloid-derived suppressor cells (MDSCs), tumor-associated macrophages (TAMs), regulatory T (Treg) cells, tumor-associated dendritic cells, mast cells, and type 2 natural killer T (NKT) cells, are involved in tumor immunosuppression (3–6). Due to their contribution to tumorigenesis and progression, MDSCs are recognized as the critical factor in TIME and their function exacerbates the disease. MDSCs may be the basis of tumor immunosuppression. First, although in tumor patients, one or several of the above-mentioned immunosuppressive cells are detected, MDSCs can be detected in most patients. Second, MDSCs suppress the innate immune response and delay the adaptive immune response. Third. MDSCs can induce expansion of immunosuppressive cells (TAMs and Treg cells).

MDSCs, have high heterogeneity, were first referred around 30 years ago and have unique characteristics and an important place in many diseases, especially cancer (7). MDSCs are a group of innate immune cells derived from myeloid lineage at different developmental stages with strong heterogeneity, which can differentiate into dendritic cells (DCs), macrophages and granulocytes under physiological conditions. However, under pathological conditions, like inflammation, trauma, tumors and autoimmune diseases, release of immunosuppressive factors block the differentiation of myeloid progenitors, promote their expansion, and further recruit them to the blood, spleen, liver, and tumor tissue (7–9). MDSCs play a pivotal and central role in governing and maintaining the TIME in solid tumors (10). MDSCs are composed of the myeloid cells with similar biological activities, but distinct phenotypes. Unlike monocytes, macrophages and DCs, which express specific molecular markers on the cell surface, MDSCs are composed of a mixture of granulocytes and monocytes, without clear and specific markers on their surface (11). In mice, MDSCs are defined as cells that co-express myeloid antigens Gr-1 and CD11b (CD11b^+^Gr-1^+^). In addition, according to the expression of Ly6C and Ly6G, CD11b^+^Gr-1^+^ cells can be further divided into granulocytic MDSCs (CD11b^+^Ly6G^+^Ly6C^low^, G-MDSC) and monocytic MDSCs (CD11b^+^Ly6G^-^Ly6C^high^, M-MDSC) subtypes (9, 12). MDSCs induced by human solid tumors were divided into two subgroups: CD33^+^HLA-DR^low^HIF1α^+^/STAT3^+^ and CD11b^+^HLA-DR^low^C/EBPβ^+^, according to their phenotypes and molecular mechanisms impeding other immune cells (13). Human monocytic MDSCs (M-MDSCs) were defined as CD11b^+^CD14^+^HLADR^-/low^CD15^-^, while granulocytic MDSCs (G-MDSCs) were defined as CD11b^+^CD14^-^CD15^+^ or CD11b^+^CD14^-^CD66b^+^ (14, 15).

In recent years, it has been discovered that MDSCs directly participate in the promotion of tumor progression and metastasis and are closely related to the clinical treatment of malignant tumors. In this review, we describe the functional and regulatory mechanism of MDSCs within TME. Notable clinical success in tumor immunotherapies, such as immune checkpoint blockade (ICB), and adoptive cell therapy (ACT), have reinvigorated our interest in the field of immunotherapy and established it as a mode of tumor therapy along with traditional strategies, like surgery, chemotherapy and radiotherapy (16, 17). Therefore, we will discuss the emerging data associated with the therapeutic strategies that targeting MDSCs. Furtherly, we also highlight what the aspects of MDSCs requires an in-depth understanding to discriminate and evaluate reasonable and sensitive combinatorial strategies to increase the efficacy of tumor immunotherapy for cancer patients.

## 2 Mechanisms of MDSC-mediated immunosuppression within TME

### 2.1 Signaling pathways related to MDSCs generation and function

**Figure 1:**
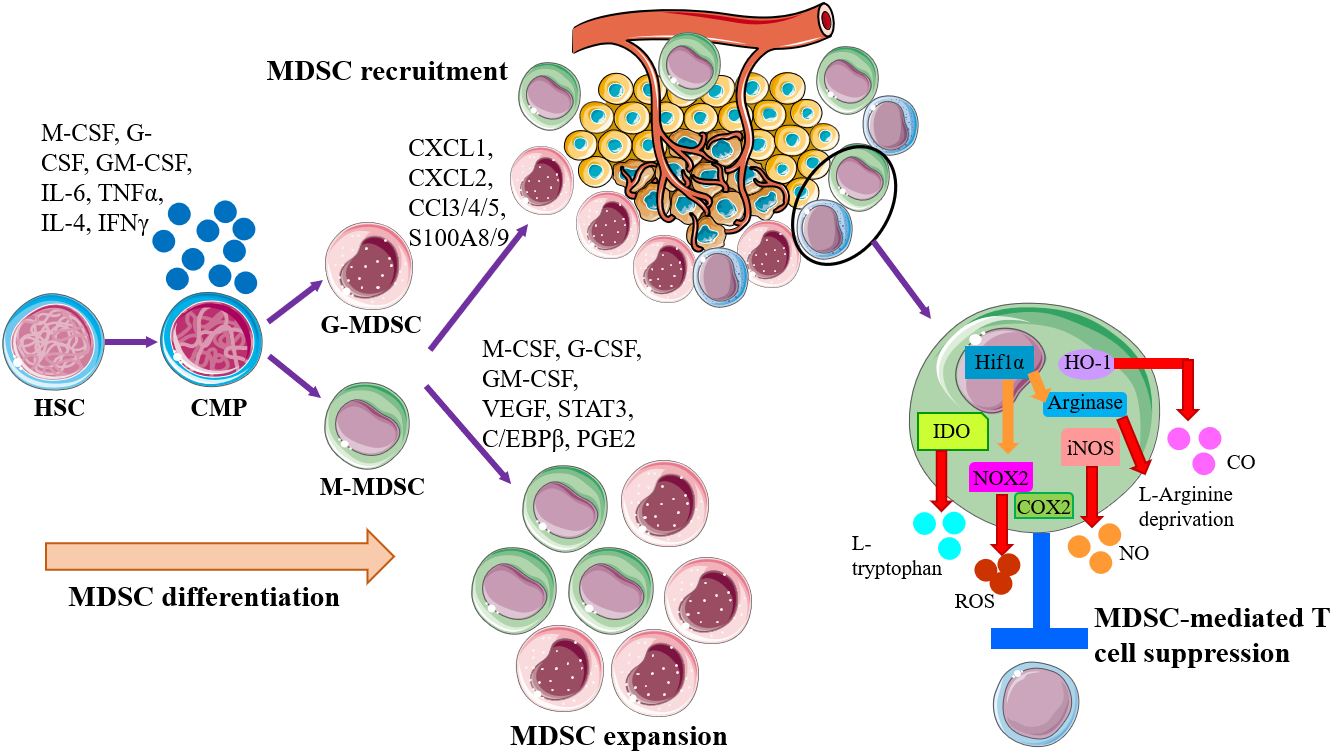
Schematic of MDSC generation, expansion, recruitment, and role in the establishment of tumor immune inhibitory microenvironment. MDSCs are generally generated by common myeloid progenitor (CMP) in bone marrow, governed by the abnormal or pathological signals, especially proinflammatory factors. Then MDSCs are recruited to tumor site by tumor-derived factors or inflammatory signals for establishing the microenvironments that promoting tumor cell escape. Within the tumor microenvironment (TME), both monocytic-MDSC (M-MDSC) and granulocytic-MDSC (G-MDSC) will expand and exert immunosuppressive functions to induce T cell suppression and anergy *via* multiple mechanisms, like arginase1, iNOS, IDO, HO-1, and NOX2.

In the setting of cancer, MDSCs can be generated by common myeloid progenitor (CMP) in bone marrow, recruited to tumor site and expand massively by tumor-derived factors or inflammatory signals, including inflammatory cytokines, chemokines, growth factors, and other pathological mediators accelerate the expansion and recruitment of immature myeloid cells to tumor site to suppress the host antitumor response (**Figure 1**). Classical ideas propose that the direct immunosuppressive function of MDSCs depends on secreting inhibitory factors, including production of nitric oxide (NO), elimination of key nutritional factors by depleting L-arginine (*via* arginase1), sequestering L-cysteine, or decreasing local tryptophan levels due to the activity of indole amine 2,3 dioxygenase (IDO) (18–21) (**Figure 1**). The mechanisms of immunosuppression by G-MDSCs and M-MDSCs are distinct to the tumor site. Tumor-mediated G-MDSC mainly inhibited T cells *via* reactive oxygen species (ROS), whereas M-MDSC inhibited T cell mainly through arginase and inducible NO synthase (iNOS) (22–25). The deprivation of L-Arginine, catabolized by MDSC-secreted Arg-1, restrained T cells proliferation *via* disrupting the expression of CD3ξ chain (19, 26). Nitric oxide (NO) is synthesized by NOSs, which are ubiquitously expressed in MDSCs, and induce T cell apoptosis by blocking JAK/STAT/nuclear factor kappa-B (NF-κB) signaling pathway (27). Peroxide nitrate (PNT) is produced by MDSCs and inhibits CD8^+^ T cells migration through reducing the integration of MHC I molecules with antigenic peptides on tumor cells and nitrate chemokines (28, 29). In response to a variety of growth factors and cytokines, the progenitor of MDSCs drive a complex transcription network, allowing for their expansion and preventing the further differentiation. The abovementioned factors trigger multiple signaling pathways in MDSCs (11, 30), and most of them converge on the activation of signal transducer and activator of transcription Janus kinase/signal transducer and activator of transcription (JAK/STAT) signaling, which upregulates immunosuppressive mediators such as iNOS, ROS, and arginase (31). STAT3 activation promoted the accumulation of MDSCs in melanoma (32), and STAT3 inhibition weakened the suppressive function of MDSCs (33). The MDSCs amplification and function are related to the downstream signals of STAT3. The calcium-binding pro-inflammatory protein factors S100A9 and S100A8, upregulated by STAT3 activation, could block the differentiation and maturation of dendritic cells (DC) and promote MDSCs accumulation (34). The exact mechanism of this process remains to be explored, but some scholars have pointed out that this may be related to the S100A9 and S100A8 heterodimers participating in the formation of the nicotinamide adenine dinucleotide phosphoric acid (NADPH) oxidase complex, which increasing the production of ROS in myeloid cells. STAT1 is the main transcription factor under IFN-γ or IL-1β stimulation, which is believed to have an important relationship with the activity of iNOS and arginase. Some studies point out that MDSCs lacking STAT1 are unable to suppress T cell function due to decreased secretion of iNOS and lower expression of arginase (35). STAT6 activated by IL-4 and IL-13 enhances the activity of arginase and inducing transforming growth factor β (TGFβ) production by MDSCs through IL-4Rα (36). Besides, Nuclear factor (NF-κB), prostaglandin E2 (PGE2)/cyclooxygenase 2 (COX2), and Ras were also of the great significance in the molecular mechanism of suppressing T cell activity mediated by specific subgroups of MDSCs (37, 38). MDSCs are affected by both novel anti-cancer immune therapies, as well as the conventional treatments such as radiotherapy. Following radiotherapy, cytoplasmic double stranded DNA stimulates the cyclic GMP-AMP synthase (cGAS)/stimulator of interferon genes (STING) pathway, resulting in type I interferon production (39). cGAS/STING signaling becomes a key factor in inhibiting MDSC function after radiotherapy *via* multiple mechanisms. The treatment of cGAMP, the STING agonist, prevented MDSC immunosuppressive function *via* reducing NO in B16 melanoma tumor-bearing mice (40). Furthermore, STING agonist treatment combined with the STAT3 inhibitor and markedly regressed tumor growth in syngeneic mice by increasing CD8^+^ T cells and Tregs and MDSCs in TME (41). Collectively, cGAS/STING and JAK/STAT pathway are both recognized as the central signaling pathway in controlling MDSC generation, accumulations and function in tumor progression. The rationale combinatorial treatment of STAT inhibitors and STING agonists will be potential therapeutic strategy and make advances in tumor immunotherapy.

### 2.2 Interplay between MDSCs and other immunosuppressive cells

Another major mechanism mediating immunosuppression is the induction and recruitment of other regulatory cells, like Treg cells (42). The characteristic of the TME enable crosstalk between MDSCs and Tregs that allows them to modulate each other mutually. MDSCs in TIME selectively facilitated expansion and induction of Treg cells *via* a TGFβ-dependent manner (43), or dependent on MDSC-secreted IL-10 and IFN-γ (23). Furthermore, MDSCs can provide additional signals for Treg cell induction and development *via* upregulating ligands expressed on the surface of MDSCs for several costimulatory molecules, such as CD86, programmed death ligand (PD-L1), and leukocyte immunoglobulin­like receptor subfamily B (LILRB4). MDSCs promoted the induction and expansion of tumor-specific Treg cells *via* taking in tumor antigens and presenting them to T cells, also converted T cells in other differentiated states into Treg cells to assist tumor evasion (44, 45). The correlation of arginase-1 expression with increasing expression of immune checkpoint receptor and ligands results in more potent suppressive activity of MDSCs (46). Twofold repression caused by MDSCs and Treg cells will create strong immune tolerance and promote tumor progression and propagation.

Thus, there is a consensus that the TME can induce MDSCs with the more potent suppressive activity *via* increasing the expression of a series of immunosuppressive molecules. Exploring more suitable approaches to blockade the immunosuppressive molecules expressed by MDSCs will be hopeful to disrupt the immunosuppression mediated by MDSCs in TME.

## 3 Current approaches and strategies targeting MDSCs for tumor immunotherapy

**Figure 2:**
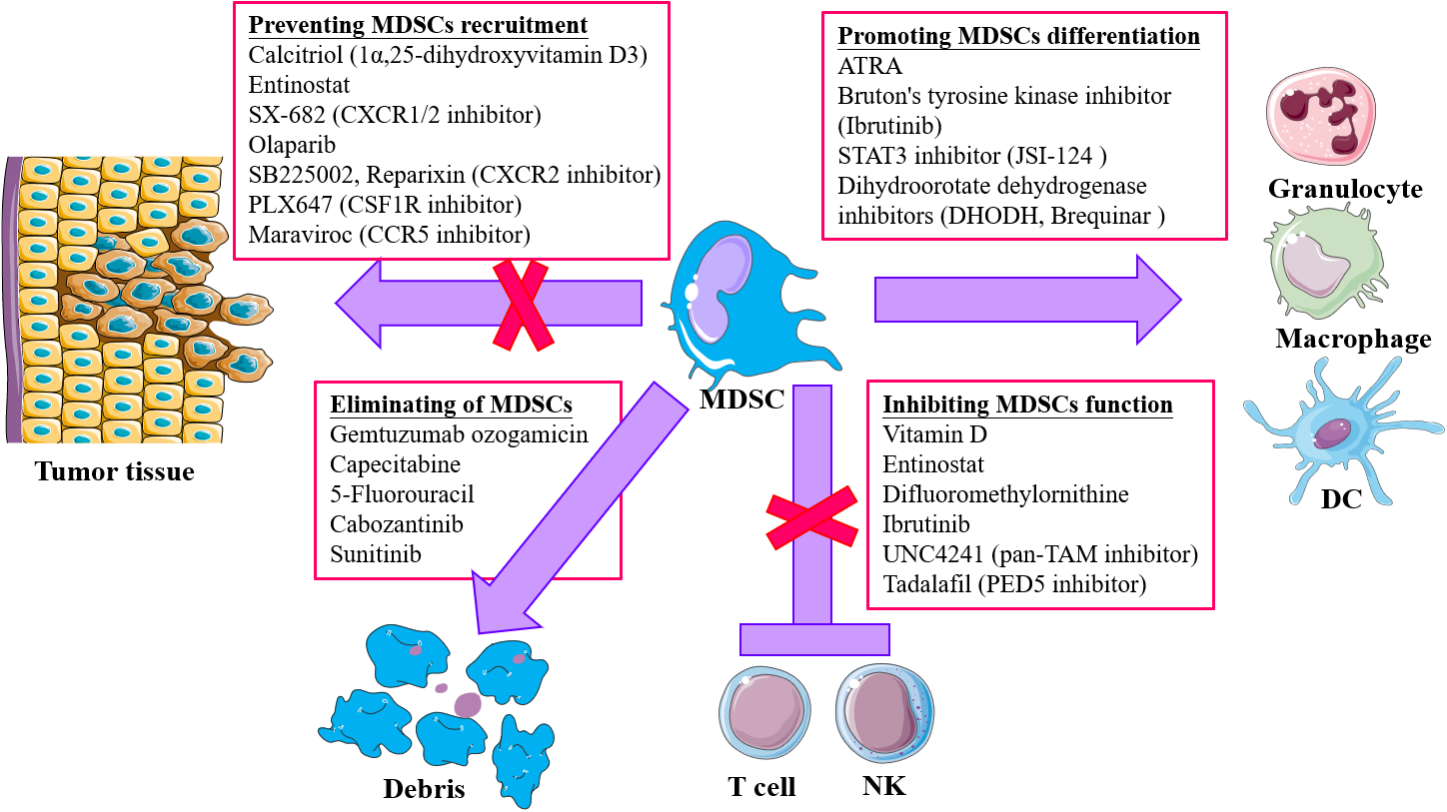
Strategies for targeting MDSCs in tumor immunotherapy. Four main approaches are included: 1) Accelerating and promoting differentiation and maturation *via* multiple agents, like all-trans retinoic acid (ATRA), STAT3 inhibitor, dihydroorotate dehydrogenase inhibitors, and Bruton’s tyrosine kinase (RTK) inhibitor; 2) blocking MDSCs recruitment and infiltration into tumor microenvironment (TME) through chemokine receptor inhibitors targeting chemokine receptors responsible for migration of MDSCs to TME; 3) depletion of MDSCs by low-dose chemotherapy and tyrosine kinase inhibitor (TKi); 4) attenuating the suppressive activity of MDSCs *via* targeting and inhibiting the effector molecules, like iNOS, COX2.

The TME plays an important role in supporting and promoting tumor growth and metastasis, where MDSCs have an important role in immuno-suppression. More studies are trying to explore and achieve tumor therapy by changing the TME (soil) to prevent the activity of tumor cells (seeds). Targeting the TIME has become a new approach for tumor therapy in recent years. In view of the important role of MDSCs in the TIME, therapeutic strategies targeting MDSCs are being explored (**Table 1**): 1) promoting the differentiation and maturation of MDSCs; 2) inhibition of the expansion and accumulation of MDSCs; 3) elimination of MDSCs in TME; 4) abolition of MDSCs immunosuppression (**Figure 2**).

**Table 1:** The therapeutic strategies of targeting MDSCs in preclinical cancer trails.

Strategy	Drug	Combinatorial partner	Tumor model		Mechanism	References
**Promoting differentiation of MDSCs**	ATRA	DC101 (antibody targeting murine VEGFR2)	The syngeneic models of breast cancer, 4T1 and TS/A		Blockade of the antiangiogenic therapy-induced expansion of MDSC secreting high levels of vessel-destabilizing S100A8	(47)
	Ibrutinib (BTK inhibitor)	None	The orthotopic mouse breast cancer model		To promote MDSCs develop into mature DCs	(48)
	Dihydroorotate dehydrogenase inhibitors (DHODH)	PD-1 inhibitor	The metastatic TNBC models, 4T1 and E0771.ML-1		To facilitate MDSCs maturation and differentiation	(49)
	JSI-124 (STAT3 inhibitor)	Sialidase	In mice bearing two transplantable tumors (EL4, CT26) and two transgenic tumors (Ret melanoma and TRAMP prostate carcinoma)		To control the differentiation of MDSC into macrophage *via* decreasing STAT3 activity	(50)
	VSSP	Anti-TLR4/anti-TLR2 mAb	Mice bearing MCA203 or the tumor-bearing mouse model using the G-CSF-producing 4T1 cell line		To induce MDSC differentiation to DC or macrophage	(51, 52)
**Inhibiting MDSCs generation, recruitment and trafficking**	Calcitriol (1α,25-dihydroxyvitamin D3)	None	The ectopic mouse tumor implantation model, CE81T and TE2		Inhibiting IL-6 signaling	(53)
	Entinostat	5-azacytidine	The NSG mice were transplanted subcutaneously of LLC tissue (Patient) and HNM007 tissue (Patient)		Downregulation of CCR2 and CXCR2, and promoting MDSC differentiation into a macrophage-like phenotype	(54)
	SX-682 (CXCR1/2 inhibitor)	CAR-NK	The mice bearing murine oral cancer 2 or cells from HNSCC patients *in vitro* or the MOC1 oral carcinoma and LLC mouse tumor models		To abrogate MDSC accumulation and trafficking, and enhance adoptive transferred NK tumor infiltration	(55, 56)
	Olaparib	EGFRvIII-targeted CAR-T	4T1EGFRvIII tumor-bearing mice		To inhibit MDSC migration *via* the SDF1α/CXCR4 axis	(57)
	JBSNF-000088 (NNMT inhibitor)	None	The xenografted tumor models overexpressing NNMT GBC cells		To inhibit MDSCs generation by decreasing IL-6 and GM-CSF expression on a epigenetic modified manner	(58)
	Icariside II	α-PD-1 mAb	LLC tumor-bearing mice		To suppress the chemotactic migration of MDSCs by downregulating the expression of CXCL2 and CXCL3	(59)
	SB225002 (CXCR2 inhibitor)	JNJ-40346527 (CSF1R inhibitor)	LLC, CT26, EL4, or 4T1 tumor-bearing mice		To block G-MDSCs infiltration and decrease TAMs	(60)
	PLX647 (CSF1R inhibitor)	Indoximod/D-1MT (IDO inhibitor)	B16-IDO tumor-bearing mouse model		To block tumor infiltrating MDSCs	(61)
	Maraviroc (CCR5 inhibitor)	anti-PD1 mAb	4T1 and PyMT breast tumor model or from patients with gastric cancer *in vitro*		To result in strong reductions of MDSCs *via* targeting autocrine CCL5-CCR5 axis	(62, 63)
	Trametinib (MEK1/2 inhibitor)	αPD-1-supplementation	4NQO-L- and B16-bearing mice		To reduce the abundance of CSF-1R^+^CD11c^+^ MDSC populations	(64)
**Preventing suppressive activity of MDSCs**	Vitamin D	None	CLL cells from patients *in vitro*		Downregulating MDSC function as negative regulator of miR155	(65)
	Entinostat	anti-PD1 mAb	The murine models of lung and renal cell carcinoma		Inhibition of immunosuppressive function of G- and M-MDSC populations by reducing arginase-1, iNOS and COX-2 levels	(66, 67)
	UNC4241 (pan-TAM inhibitor)	α-PD-1 mAb	Melanoma tumor-bearing mice		To diminish MDSC suppression and differentiation in part through regulation of STAT3 serine phosphorylation and nuclear localization	(68)
	Difluoromethylornithine	None	B16 tumor-bearing mice		Inhibition of ODC by DFMO is to impair MDSCs suppressive activity *via* reducing arginase expression and inhibiting the CD39/CD73-mediated pathway	(69)
	Ibrutinib (BTK inhibitor)	Anti-PDL1 checkpoint inhibitor	Neuroblastoma tumors- bearing mice		To alter NO production, and decrease expression of IDO, Argnaise, TGFβ	(70)
**Elimination of MDSCs**	Gemtuzumab ozogamicin	CAR-T	NSCLC; PA; BIDC; CA; PDA cell lines *in vitro*		To deplete MDSCs for reactivating CAR-T cell responses against multiple cancers	(71)
	5-Fluorouracil or capecitabine (5-FU pro-drug)	Gemcitabine	EL4-bearing mice or pancreatic cancer patients		To eliminate MDSCs *via* selectively induce MDSCs apoptosis	(72, 73)
	MD5-1 (anti-DR5 antibody)	Anti-PD-L1 antibody			To deplete MDSCs and induce enrichment of CD8^+^ T cells	(74)
	Cabozantinib	Anti-HER2 mAb	4T1-HER2 murine breast cancer model		To delete MDSCs and improve the efficacy of anti-HER2	(75)
**Decreasing immune checkpoint receptors expression on MDSCs**	Anti-CD200 mAb	Anti-PD-1 antibody	MT-5 tumor-bearing mice and genetically engineered PDAC mouse model		To limit CD200R^+^ MDSCs expansion	(76)
	Anti-gp49B (LILRB4) antibody	Anti-PD-1 antibody	LLC-tumor bearing mice		To decrease M-MDSCs infiltration	(77)
	HMBD-002 (anti-VISTA antibody)		CT26, HCT15, A549, and 4T1 tumor-bearing		To decrease the infiltration of MDSCs and increase T cell activity	(78)

ATRA, all-trans retinoic acid; BTK, bruton’s tyrosine kinase; TNBC, triple-negative breast cancer; VSSP, very small size proteoliposomes; RCA, renal cell carcinoma; DCs, dendritic cells; LLC, lewis lung carcinoma; ESCC, esophageal squamous cell carcinoma; MOC2, murine oral cancer 2; CLL, chronic lymphocytic leukemia; PDAC, pancreatic ductal adenocarcinoma; TMA RTK, transmembrane receptor tyrosine kinases; ODC, ornithine decarboxylase; DFMO, difluoromethylornithine; mAb, monoclonal antibody; NSCLC, non-small cell lung carcinoma; PA, prostate adenocarcinoma; BIDC, breast invasive ductal carcinoma; CA, colon adenocarcinoma; PDA, pancreas duct adenocarcinoma; NNMT, nicotinamide N-methyltransferase; GBC, gallbladder carcinoma; CXCL2, CXC chemokine ligands 2; CXCL3, CXC chemokine ligands 3; ICB, immune checkpoint blockade; HNC, head and neck cancer; IDO, indoleamine 2,3-dioxygenase; APC, advanced pancreatic cancer; 5-FU, 5-Fluorouracil; LILRB4, leukocyte immunoglobulin-like receptor subfamily B member 4; VISTA, V-domain Ig suppressor of T cell activation.

### 3.1 Therapies promoting differentiation of MDSCs into mature cells

MDSCs are a mixture of immature myeloid cell populations with high heterogeneity and immunosuppressive activity. All-trans retinoic acid (ARTA) could promote the differentiation of MDSCs into granulocytes, macrophages and DC, improve the host anti-tumor immune response *via* neutralizing the production of ROS (79–81). For example, the administration of formic acid receptor (RAR) antagonist (which does not affect retinol X receptor (RXR)) in mice can cause the accumulation of granulocytes in various hematopoietic organs, including bone marrow, suggesting that the RAR pathway blocks the differentiation and maturation of granulocyte precursors. 1,25-dihydroxy vitamin D3 (1,25(OH)2D3), which is the active metabolite of vitamin D3, has been identified as a potent natural modulator of innate and adaptive immunity. Vitamin D3 combined with various cytokines induced the differentiation of CD34^+^ progenitors isolated from patients with head and neck squamous cell carcinoma (HNSCC), resulting in increased numbers of cells phenotypically similar to mature DCs (82). Recent studies have provided important insights that primitive myeloid leukemic cell lines can be driven to differentiate into monocyte-like cells by 1,25(OH)2D3, which may be useful in differentiation therapy of myeloid leukemia and myelodysplastic syndromes (MDS) (83, 84). However, the role of vitamin D3 in myeloid cell differentiation remains controversial. A recent study showed that DCs treated with 1α,25(OH)2D3 (calcitriol) did not differentiate or mature, locking the cells in a tolerogenic/immature state (85). Vitamin D3-induced tolerogenic DCs are thought to develop their regulatory properties through a semimature profile, inhibition or reduction of T-cell responses, and switching the immune response to a Th2 profile (86–89). Vitamin-D3-induced tolerogenic DCs with the semimature phenotype, anti-inflammatory profile, and low capacity to induce T-cell proliferation, can be used clinical for inducing immunotolerance (90). Calcitriol attenuated the recruitment of MDSCs and increased infiltration of cytotoxic T cells following radiotherapy in hepatocellular carcinoma and prostate cancer (82, 91). Thus, the role of vitamin D3 in tumor therapy is complex and the application of vitamin D3 for clinical use by targeting MDSCs still needs more study. By promoting the development of MDSCs into normal monocytes and granulocytes, not only reduces MDSCs, but also increases the mature myeloid cells in TIME, thereby inhibiting tumor growth.

### 3.2 Strategies that inhibiting the expansion and recruitment of MDSCs

As mentioned above, the expansion of MDSCs is regulated by tumor-derived suppressive factors secreted by tumor cells and released by the TME. It mainly includes IL-6, GM-CSF, G-CSF, VEGF, COX-2 and other cytokines, which can trigger a variety of different signal transduction and signal activation pathways in MDSC. The STAT3 signaling pathway is an important regulator for MDSC amplification mediated by these factors and could be the ideal target. STAT protein has an N-terminal DNA-binding domain and C-terminal protein-binding domain. Tumor-derived suppressive factors binding to corresponding receptors leads to continuous activation of STAT3, which then upregulates expression of STAT3-related genes and produces proteins (survivin and cyclin) and matrix metalloproteinase-9 (MMP-9) that promote MDSCs expansion. Targeting the STAT3 signaling pathway has become a research hotspot in the inhibition of MDSC expansion (92). MMP-9 is an important target for tumor therapy *via* inhibiting amplification of MDSCs and facilitating formation of the TME. MMP-9 inhibitors promote the normalization of hematopoietic function, thus reducing the production of MDSCs in tumor-bearing BALB-neuT mice expressing an activated rat c-erbB-2/neu transgene model (93). VEGF is currently recognized as the most powerful angiogenic factor, which can specifically act on vascular endothelial cells and promote vascular endothelial hyperplasia. Studies have confirmed that VEGF in tumor tissues can promote the generation of neovascularization, inhibit the development of DCs and induce the generation of MDSCs (94, 95). Therefore, blockade of VEGF could be another approach for tumor therapy, by removing immunosuppression. In a tumor-bearing mouse model, tumor growth was significantly inhibited after administration of anti-VEGF antibody, and there was a reduced number of MDSCs in tumor tissue and peripheral blood. However, the mechanism by which anti-VEGF monoclonal antibody (mAb) inhibits the expansion of MDSCs remains to be elucidated (96, 97). Bevacizumab was the first FDA-approved monoclonal antibody against VEGF, which brings hope to patients with advanced tumors by anti-tumor microangiogenesis and inhibiting the progression of metastatic lesions. Although, the application of anti-VEGF mAb has been verified and evaluated in a multitude of clinical trials for tumor therapy, because of the multiple effects mediated by blocking VEGF, the efficacy could not be only attributed to MDSC reduction. Sunitinib, as the anti-angiogenic drug, is a receptor tyrosine-kinase inhibitor and immunomodulator, that potently prevents MDSC accumulation and restores normal T-cell function in tumor-bearing mice, independent of its capacity to inhibit tumor progression, as well as reverses MDSC accumulation and T-cell inhibition even in the blood of non-responder renal cell carcinoma (RCC) patients (98).

Chemokines as key mediators of MDSCs recruitment have been extensively studied in many tumor models and cancer patients (**Table 1**). The recruitment of MDSCs from bone marrow and spleen to tumor tissues is mainly through multiple signaling pathways. A pivotal role of CCL2-CCR2 signaling in MDSC recruitment and tumor progression has been demonstrated in melanoma and hepatocellular carcinoma (HCC) mouse models (99, 100). Thus, blocking CCL2 with soluble CCR2 fragment or inhibition of CCR2 with blocking antibody could decrease tumor accumulation of MDSCs in the tumor bone metastases model by injecting prostate cancer cells directly into murine tibiae, making it a potential target for anti-tumor therapy (101). mCCR5-Ig fusion protein, anti-CCR5 antibody, or even CCL5-neutralizing mAb were all found that could reduce the number and suppressive capacity of tumor infiltration MDSCs, prevent the tumor metastasis, promote the survival of B16 tumor-bearing mouse and even improve the efficacy of anti-PD-1 tumor therapy (102, 103). Chemokine (C-X-C motif) ligand 8, also known as IL-8, highly expressed in various tumors, including colon, ovarian, breast, pancreatic, prostate, and hematological malignancies (104–106), has been demonstrated that could recruit MDSCs to tumor sites *via* CXCR1/CXCR2 (107). The treatment of Reparixin, the pharmacological inhibitor of CXCR1 and CXCR2, caused the significant reduction of G-MDSCs numbers, in colon adenocarcinoma HT29 xenograft tumor and colon carcinoma CT26-GM-derived subcutaneous tumor models (108–110). Furthermore, inhibition of MDSC trafficking by SX-682, a CXCR1/2 inhibitor, enhanced NK-Cell immunotherapy in head and neck cancer models (55). HuMax-IL8, an anti-IL-8 mAb, reduced the number of G-MDSCs in MDA-MB-231 breast cancer xenografts (111). CXCL8 levels determine the efficacy of sunitinib treatment, which was demonstrated to effectively target MDSCs, suggesting that CXCL8 acts as a potential target in anti-tumor therapy (112). Blocking MDSC recruitment to tumor tissues may be an effective approach for disrupting the formation of TIME and improving the efficacy of anti-tumor therapy.

### 3.3 Strategies abolishing MDSC immunosuppression

MDSCs mainly express reactive ROS, arginase1, NOS and peroxynitrite to exert their immunosuppressive function (28, 113). Therefore, appropriate inhibition of those factors, serving as important potential therapeutic targets, can eliminate the immunosuppression of MDSCs. ROS, as part of the major mechanism by which MDSCs suppress T-cell responses, activate anti-oxidative pathways and induce transcriptional programs that regulate the fate and function of MDSCs. Nuclear factor erythroid 2-related factor 2 (Nrf2) activation in regulating the constitutive activation and availability of antioxidant enzymes, including NADPH, NADPH quinone oxidoreductase 1 (NQO1), hem oxygenase (HO), might be a central mechanism enabling cells to increase mitochondrial ATP production by simultaneously counteracting subsequent high ROS levels (114). Selective activation of Nrf2 can decrease the intracellular ROS production, inhibit the immunosuppression of MDSCs, prevent tumor metastasis, and induce tumor regression. Furthermore, the synthetic triterpenoid C-28 methyl ester of 2-cyano-3,12-dioxooleana-1,9,-dien-28-oic acid (CDDO-Me) completely abolished the immunosuppressive activity of MDSCs by reducing ROS production in mouse tumor models (115). Moreover, the treatment of pancreatic cancer patients with CDDO-Me did not affect the number of MDSCs in peripheral blood of patients but significantly improved the immune response (115). Agents that target MDSCs, such as sanguinarine (SNG), are now being considered for treatment of lung cancer. SNG was found to inhibit the immunosuppressive activity of MDSCs *via* decreasing the expression of Arg-1, iNOS, and ROS, as well as inducing the differentiation of MDSCs into macrophages and DCs through the NF-κB pathway *in vitro* from Lewis lung cancer mouse model (116). The type I interferons pathway is well known to promote anti-tumor immunity by diverse mechanisms. Emerging evidence shows that the downregulation of the IFNAR1 chain is found in MDSC from cancer patients and mouse tumor models. The decrease in IFNAR1 depends on the activation of the p38 protein kinase and is required for activation of the immunosuppressive phenotype (117). Stabilizing IFNAR1 using p38 inhibitor combined with IFN induction therapy elicits a robust anti-tumor effect *via* undermining suppressive activity of MDSCs in tumor bearing mice (117). The JAK/STAT signaling pathway is one of the well-known pathways induces immune escape of tumors *via* cytokines and growth factors to control MDSC generation and differentiation (118, 119). Blockade of STAT3, STAT5 or even NF-κB by the selective inhibitors can inhibit the immunosuppression of MDSCs (120). AMP-activated protein kinase α (AMPKα) signaling was increased in tumor-MDSCs from tumor-bearing mice and patients with ovarian cancer, which was induced by tumor-derived GM-CSF and occurred in a STAT5-dependent manner (121). In addition, genetic deletion of ampkα1-coding gene, prkaa1 antagonized M-MDSC differentiation to macrophages and re-routed M-MDSC, but not G-MDSC, into cells that elicited direct antitumor cytotoxic effects through NOS2-mediated actions, suggesting the therapeutic use of AMPK inhibitors to overcome MDSC-induced T-cell dysfunction and AMPK inhibition as a potential therapeutic strategy to restore protective myelopoiesis in cancer. G-MDSCs in the TME spontaneously die by ferroptosis, inducing the release of oxygenated lipids and limiting the activity of human and mouse T cells, although decreasing the presence of G-MDSCs (122). Thus, genetic and pharmacological inhibition of ferroptosis by liproxstatin-1, abolishes suppressive activity of G-MDSCs, reduces tumor progression and synergizes with immune checkpoint blockade (ICB) to suppress tumor growth in immunocompetent mice (122). However, induction of ferroptosis in immunocompetent mice promotes tumor growth. Therefore, ferroptosis is a unique and targetable immunosuppressive mechanism of G-MDSCs in TME that can be pharmacologically modulated to limit tumor progression. In human hepatocellular carcinoma (HCC), the tumor-surrounding fibrotic livers were markedly enriched with M-MDSCs, along with the poor survival rates. Mechanistically, activated hematopoietic stem cell (HSC) induced monocyte-intrinsic p38 mitogen-activated protein kinase (MAPK) signaling to trigger enhancer reprogramming for M-MDSC development and immunosuppression (123). Treatment with p38 inhibitor inhibited HSC-M-MDSC crosstalk to prevent HCC growth (123). Concomitant with patient-derived M-MDSC suppression by i-BET762, combined treatment with anti-PD-L1 synergistically enhanced tumor-infiltrating lymphocytes, resulting in tumor eradication and prolonging survival in the fibrotic-HCC mouse model (123). It has been reported that mouse and human G-MDSCs exclusively upregulate fatty acid transport protein 2 (FATP2), which was controlled by GM-CSF, through activation of the STAT5 (124). The selective pharmacological inhibition of FATP2 abrogated the suppressive activity of G-MDSCs and substantially delayed tumor progression (124). In combination with immune checkpoint inhibitors (ICIs), FATP2 inhibition blocked tumor progression in mice and has the potential to improve the efficacy of cancer therapy.

### 3.4 Therapies eliminating MDSC within TME

An initial attempt was made to clear the MDSCs with antibodies against Gr-1, but since Gr-1 is not specifically expressed by MDSCs, which is also expressed by mature granulocytes. Therefore, the elimination of MDSCs may also lead to a decline in normal immune cells. In addition, once the plasma concentration of the antibody in plasma decreases or the immune system responds to the antibody, the number of MDSCs increases rapidly, which enhances the immunosuppressive function of MDSCs in the TME. Low dose of chemotherapy, such as 5-fluorouracil (5FU), paclitaxel, cisplatin and gemcitabine, has been shown to effectively eliminate MDSC in tumor-bearing mice, and enhanced anti-tumor immunity (72, 73, 125–127). The number of MDSCs in the spleen was significantly reduced, although DCs, T cells, NK cells, macrophages and B cells were not significantly affected. The mechanism may be that the chemotherapy drugs belong to base analogs, which can prolong and block the DNA synthesis in the cell cycle and induce cell death. However, the mechanism of selective killing of MDSCs needs to be further clarified. Additionally, subclinical doses of platinum-based drugs, such as cisplatin, prevented the generation and suppressive activity of M-MDSCs by inhibiting STAT3-COX2 signaling pathway, along with decreasing COX2 and arginase1 expression in M-MDSCs of melanoma and head and neck squamous cell carcinoma (HNSCC) patients (128). Therefore, some chemotherapy drugs can play an active role in anti-tumor immunotherapy by targeting MDSCs in a certain dosage and course of treatment.

The remodeling of metabolic states also contributes to the shape of the TIME and plays an important role in regulating MDSCs in the TME. The radiotherapy-augmented Warburg effect helps myeloid cells to acquire an immunosuppressive phenotype, resulting in limited treatment efficacy for pancreatic ductal adenocarcinoma (PDAC) (129). Sustained increase in lactate secretion, resulting from the radiation augmented Warburg effect, was responsible for the enhanced immunosuppressive phenotype of MDSCs after radiotherapy (129). Thus, targeting lactate derived from tumor cells and the hypoxia-inducible factor-1α (HIF-1α) signaling in MDSCs could reinstate antitumor T-cell responses and inhibit tumor progression after radiotherapy in pancreatic cancer, indicating distinct promise for clinical therapies to alleviate radio resistance in PDAC. Glutamine metabolism is a crucial element of cancer cell metabolism. Glutamine is important for nucleotide synthesis, amino acid production, redox balance, glycosylation, extracellular matrix production, autophagy, and epigenetics (130, 131). Emerging evidence shows that targeting tumor glutamine metabolism leads to a decrease in G-CSF and hence recruitment and generation of MDSCs as well as immunogenic cell death, leading to an increase in inflammatory TAMs (132). Alternatively, inhibiting glutamine metabolism of the MDSCs themselves not only led to activation-induced cell death and conversion of MDSCs to inflammatory macrophages, also impaired suppressive function of MDSCs *via* inhibiting IDO expression in the tumor and MDSCs, that resulted in a marked decrease in kynurenine levels, and rendered checkpoint blockade-resistant tumors susceptible to immunotherapy in tumor-bearing mice (132). Therefore, the application of glutamine antagonism in synergistic targeting inhibition of tumor and MDSCs may hold promise for clinical therapy to inhibit tumor growth and metastasis. Therapeutic liver-X nuclear receptor (LXR) agonism was also found to reduce MDSC abundance in murine models and in patients treated in a first-in-human dose escalation phase 1 trial, accompanied with the activation of cytotoxic T lymphocyte (CTL) responses in mice and patients (133).

## 4 significance of tumor immunotherapy combined with MDSC-targeted therapies

### 4.1 Combination of MDSC-targeted therapies with adoptive cell therapy

Tumor immunotherapy, such as ICB and adoptive cell therapy, has attracted much attention in recent years due to its remarkable efficacy. However, preliminary and limited success is achieved in hematological malignancies and in certain solid tumors, owing to the limitations in curative effect, or in technology. Combined treatment strategy is suggested which may improve the efficacy of mono-immunotherapy and compensate for the deficiency of monotherapy. MDSCs mediate tumor metastasis and are implicated in immune evasion through shaping the TME, and are referred as the “queen bee” of the TME (134). Strategies to reverse the suppressive TME should also attract and activate immune effectors with antitumor activity (**Table 2**). Cytokine-induced killer (CIK) cell-based immunotherapy is effective as adjuvant therapy in HCC with early stage but lacks efficacy in advanced HCC. MDSCs are increased in response to CIK cell therapy and subsequently may be targeted to provide an additional therapeutic benefit. A study on immunosuppressive mechanisms focusing on CIKs found that combination treatment with a PDE5 inhibitor reversed the MDSC suppressor function *via* arginase-1 and iNOS blockade and systemic treatment with a PDE5 inhibitor prevented MDSC accumulation in the TME of the tumor bearing mice (135). Similarly, treatment with a PDE5 inhibitor suppressed CD14^+^HLA-DR^−/low^ MDSCs immunosuppressive activity and enhanced CIK activity against human HCC cell lines *in vitro*, suggesting targeting MDSCs is an efficient strategy to enhance the antitumor efficacy of CIKs for the treatment of patients with HCC. The possible combination of olaparib with EGFRvIII-targeting CAR (806-28Z CAR) T cells has been explored (57). The hostile TME is also one of the major obstacles to the efficacy of chimeric antigen receptor modified T (CAR-T) cells, and the recruitment of MDSCs within the TME may contribute to the unsatisfactory performance of CAR-T cells in solid tumors. Olaparib might suppress the recruitment of MDSCs to improve the TIME, which contributes to the infiltration and survival of CAR-T cells on breast cancer in mice (57). The additional mechanistic rationale for combining the third-generation PARPi (olaparib) with CAR-T therapy for the treatment of breast cancer was supported. GPC3-CAR T cell treatment together with C1632, the inhibition of Lin28, which targets IDO1 and PDL1, led to enhanced anti-tumor activity in a HCC xenograft mouse model (136). Combination of targeting IDO1 and PDL1 with CAR-T cells serves as a dual targeting agent against tumor cells and MDSCs in TME and enhances immunotherapeutic potential of CAR-T cells against tumor.

**Table 2:** Summary of clinical trials targeting MDSCs in cancer.

Drug	Target	Combinatorial partner	Cancer	Outcome	ClinicalTrials.gov identifier
**ATRA**	Retinoic acid receptor	Ipilimumab	Melanoma	None of the patients in the Ipilimumab plus ARTA group had signs of disease progression	NCT02403778
**ATRA**	Retinoic acid receptor	Pembrolizumab	Advanced melanoma	Ongoing	NCT03200847
**HF1K16**	ATRA	None	RST	Recruiting and ongoing	NCT05388487
**Entinostat**	Class I HDAC	Azacitidine	NSCLC	Combined epigenetic therapy decreases relapses after curative surgery	NCT01207726
**Entinostat**	Class I HDAC	Clofarabine	ND ALL/ABL; R/R ALL/ABL	Entinostat plus clofarabine appears to be tolerable and active in older adults with ND ALL/ABL, but less active in R/R patients	NCT01132573
**Entinostat**	Class I HDAC	Exemestane	ER^+^ breast cancer	8.3-mo improvement in OS among patients who received entinostat	NCT02115282
**SX-682**	CXCR1/2	Pembrolizumab	Melanoma	Recruiting and ongoing	NCT03161431
**Capecitabine**	DNA/RNA synthesis	Bevacizumab	GBM	Circulating MDSCs were lower and the increased cytotoxic immune infiltration was observed after low-dose capecitabine treatment	NCT02669173
**Tadalafil**	PDE5	None	HNSCC	Significantly reducing both MDSCs and Treg and increasing CD8^+^ T cells reactive to autologous tumor antigens	NCT00843635
**Gemcitabine**	DNA/RNA synthesis	Nivolumab	NSCLC	Decreasing MDSCs to enhance anti-PD1 therapy	NCT03302247
**Omaveloxolone (RTA 408)**	Nrf2	Ipilimumab or nivolumab	Melanoma	The best overall in omaveloxolone (5 mg) & ipilimumab group is up to 100%	NCT02259231
**Dasatinib**	Tyrosine kinase	DC vaccines	Metastatic melanoma	Combined treatment was safe and resulted in coordinating immunologic and/or objective clinical responses in 6/13 (46%) evaluable patients	NCT01876212
**MTL-CEBPA**	C/EBPα	Pembrolizumab	AST	Causing inactivation of MDSCs with potent antitumor responses across different tumor models and in cancer patients	NCT04105335
**Reparixin**	CXCR2	Paclitaxel	TNBC	Weekly combinatorial treatment in MBC appeared to be safe and tolerable, with demonstrated responses in the enrolled population	NCT02370238
**RGX-104**	LXR	Nivolumab, ipilimumab, docetaxel	EC, NSCLC	Recruiting and ongoing	NCT02922764
**Tasquinimod**	S100A9	None	mCRPC	Tasquinimod significantly improved rPFS compared with placebo	NCT01234311
**Sunitinib**	VEGF and c-KIT	None	RCC	The therapy is feasible, safe and an effective method to manage toxicity in metastatic renal cell carcinoma	NCT01499121
**Aspirin**	COX2	Ipilimumab, Pembrolizumab	Melanoma	To inhibit the function of tumor MDSCs	NCT03396952
**Maraviroc**	CCR5	None	CRC	Mitigation of tumor-promoting inflammation within the tumor tissue and objective tumor responses in CRC were observed.	NCT01736813

ATRA, all-trans retinoic acid; RST, refractory solid tumors; ND ALL/ABL, newly diagnosed acute lymphoblastic leukemia/acute biphenotypic leukemia; R/R ALL/ABL, relapsed/refractory acute lymphoblastic leukemia/acute biphenotypic leukemia; NSCLC, non-Small Cell Lung Carcinoma; HDAC, histone deacetylase; ER, estrogen receptor; OS, overall survival; GBM, glioblastoma brain tumors; PED5, phosphodiesterase-5; HNSCC, neck squamous cell carcinoma; Nrf2, erythroid 2-related factor 2; DC, dendritic cell; AST, advanced solid tumor; TNBC, triple-negative breast cancer; MBC, metastatic breast cancer; EC, endometrial cancer; mCRPC, metastatic castration-resistant prostate cancer; rPFS, radiographic progression-free survival; RCC, renal cell carcinoma; CRC, colorectal cancer; COX2, cyclooxygenase-2; CXCR2, CXC chemokine receptor 2; CCR5, C-C motif chemokine receptor type 5.

### 4.2 Combination of MDSC-targeted therapies with immune checkpoint therapy

Although ICB therapy has made remarkable achievements in tumor immunotherapy, there is still a large proportion of patients that do not respond to ICIs or develop resistance (137, 138). Furthermore, ICB therapy is disappointing with a response rate < 10% in cancers with a poorly immunogenic or “cold” TIME, requiring further strategies for effective immunotherapy (139, 140). Immunotherapy non-responders often harbor high levels of circulating MDSCs which can predict the response to cancer immunotherapies, which is an important factor in developing resistance to ICB therapy and mediates immunosuppression in TME, hindering the efficacy of such therapy (141). In particular, the levels of MDSCs indicate whether the patients will respond to ICIs, which the close association between MDSCs level in patients with the efficacy of anti-PD1 or anti-CTLA4 therapy has been observed (142–144). PD-L1 is usually expressed in the majority of cancers, and PD-L1 expression by host myeloid cells is more effective than that on cancer cells in suppressing CTL function (145–149). MDSCs may also suppress CTL activity by PD-L1-dependent and -independent mechanisms (29). Therefore, combining TAM and G-MDSC inhibitors reduced both populations in the tumor site, and dramatically enhanced the effect of ICB with anti-PD-1 in our preclinical model of cholangiocarcinoma (CCA) (150). Combining ICI treatment with MDSC depletion has been successful and has been investigated in some pre-clinical studies. Combined treatment using entinostat and 5-azacytidine, epigenetic modulatory drugs, with ICB antibodies (anti-PD1 and anti-CTLA4), led to complete tumor regression and metastatic progression in the aggressive triple-negative breast cancer (TNBC) model 4T1, with >80% survival rate 100 days after tumor implantation (54, 151). IL-6 plays a major role in the accumulation and activation of MDSCs during tumor development. Importantly, increased IL-6 levels positively correlate with disease progression and MDSC enrichment in cancer patients (152). Preclinical studies of IL-6/IL-6R blockade to target MDSCs in cancer have been conducted. IL-6 blockade or anti-IL-6R monoclonal antibody reversed the effect of ICIs in HCC, colorectal cancer, melanoma, triple negative breast cancer, and squamous cell carcinoma, along with marked reduction of MDSCs, decreased suppressive activity of MDSCs, or an increase in tumor infiltrating CD8^+^ effector T cells (153–157). Only combination therapy in targeting MDSCs and immune checkpoints was more effective for anti-tumor therapy, while only using the epigenetic modulatory drugs did not mediate the anti-tumor immunity (66). Mechanistic insight into the reversibility of epigenetic modification through small-molecule inhibitors has unlocked the possibility of targeting specific epigenetic pathways to reprogramme the MDSC population into an immunostimulatory phenotype. The histone deacetylases inhibitor, entinostat, was shown to block the formation of the premetastatic niche *via* promoting MDSCs differentiation into pro-inflammatory macrophages and the therapeutic use of entinostat has been observed limited efficacy in some clinical trials (NCT01207726, NCT01132573, NCT02115282) (54). However, entinostat-driven inhibition of MDSC activity combined with ICI resulted in the tumor regression and longer tumor-free survival by improving the infiltration and function of granzymeB^+^CD8^+^ T cells in mouse models of HER2 transgenic breast cancer and the Panc02 metastatic pancreatic cancer mouse models (3, 67). The m6A demethylase Alkbh5 has effects on m6A density and splicing events in tumors during ICB therapy and modulates MDSCs accumulation in TME by regulating Mct4/Slc16a3 expression and lactate content of the TME in the employed melanoma and colon syngeneic mouse models (158). Thus, a small-molecule Alkbh5 inhibitor enhanced the efficacy of ICB cancer immunotherapy. ATRA can have positive effects on anti-tumor therapy by reprogramming MDSCs state within the TME. Some clinical trials have also demonstrated the potential significance of ATRA alone or in combination with ICB or target- orientated anticancer drug in anti-tumor therapy (NCT02403778, NCT03200847). It has been reported that addition of ATRA, which reduces expression of immunosuppressive genes including *PD-L1*, *IL-10*, and *IDO* by MDSCs, to standard of care ipilimumab appeared safe (159). Finally, ATRA significantly decreased the frequency of circulating MDSCs compared to ipilimumab alone in advanced-stage melanoma (159). Additionally, ATRA has been demonstrated to increase the efficacy of anti-VEGFR2 antibodies alone and in combination with chemotherapy in preclinical breast cancer models, reverse the anti-VEGFR2-induced accumulation of intratumoral MDSCs, alleviate hypoxia, and counteract the disorganization of tumor microvessels (47). Although, the clinical efficacy of ATRA has been evaluated, the more effective combination treatment needs to be further explored. We highlight the current clinical trials ongoing and testing the combination of targeting-MDSC with ICB, chemotherapy in **Table 2**.

### 4.3 Engineering CAR-T cells to deliver the targeting agents against MDSC

Genomic and epigenomic editing provides more opportunities for immunotherapies to create and alter properties. Furthermore, gene editing for immune cell therapies saves the cost and labor participating in the manufacture of the cell products. Gene-modified T cell therapy has been developed as a way to deliver T cells targeting different targets of tumor. However, the immunosuppressive tumor microenvironment is still one of multiple barriers existing in solid tumors that continue to hinder the efficacy of CAR-T cells. Thus, gene modification may enable CAR-T cells acting as a dual targeting agent against tumor cells and MDSCs. One study has developed a modified CAR T cells with IL15, targeting the receptor IL15 receptor alpha (IL15Rα) expressed on MDSC in human and murine glioblastomas (GBMs) (160). The fusion of IL15 to the antibody part of CAR T cells generates a dual targeting system that diminishes the frequency MDSC and tumor cells and improved the survival of mice in two GBM models (160). Another study engineered CAR-T cells to deliver RN7SL1, an endogenous RNA that activates RIG-I/MDA5 signaling (161). RN7SL1 promotes expansion and effector-memory differentiation of CAR-T cells, and transferred RN7SL1 restricts MDSC development, decreases TGFβ in myeloid cells, and fosters DC subsets with costimulatory features, which enables CAR-T cells to enhance autonomous and endogenous immune function (161). To reverse the suppressive tumor microenvironment, some study developed gene modified T-cells bearing a chimeric receptor in which activating receptor NKG2D fused to intracellular domains of 4-1BB and CD3z (NKG2D CAR) (162). The NKG2D CAR-T cells target MDSCs, which overexpress Rae1 (NKG2D ligands) within the TME. NKG2D CAR-T cells eliminated MDSCs and improved antitumor activity of subsequently infused CAR-T cells in a novel orthotopic implantation of syngeneic pancreatic ductal adenocarcinoma (PDAC) tissue slices mice model (162). Similar results were observed in the study that used gene-modified NK cells bearing a chimeric receptor in which the activating receptor NKG2D was fused to the cytotoxic ζ-chain of the T-cell receptor (NKG2D.ζ), and targeted MDSCs that overexpressed NKG2D ligands within the TME (163, 164). Confirmed in the clinical trial, NKG2D.ζ-NK cells generated from patients with neuroblastoma killed autologous intra-tumoral MDSCs capable of suppressing CAR-T function (NCT03373097) (164). Combination therapy with NKG2D.ζ-NK cells and CAR-T cells for solid tumors may provide superior efficacy compared to CAR-T cells therapy alone. PD-L1 axis is a key immunosuppressive signal provided by tumor cells and MDSCs in TME, which limits CAR-T cell function. Some studies designed the CAR-T cells secreting anti-PD-L1 single-chain variable fragment (scFv) or generated a novel PD-L1-targeting chimeric switch receptor (PD-L1.BB CSR) (165–167). The former CAR-T cells secreting anti-PD-L1 scFv which could bind to PD-L1 on PD-L1^high^ tumor cells and MDSCs competitively and block their binding with anti-PD-L1 monoclonal antibodies, leading to increased efficacy (165). The latter CAR-T cells can bind to PD-L1, switching the inhibitory signal into an additional 4-1BB signal, displayed superior fitness and enhanced functions in culture medium, causing rapid and durable eradication of pleural and peritoneal metastatic tumors in xenograft models (166). Furthermore, a phase I clinical trial related to this study, was initiated in patients with pleural or peritoneal metastasis (NCT04684459). Thus, those studies open the opportunity for investigating other targeting moieties on the surface of MDSCs, specifically those enriched in cells of TME, and applying these modifications to CAR T cells for their direct dual functions against glioma cells and immunosuppressive MDSC.

### 4.4 Combination of MDSC-targeted therapies with tumor vaccine

Recent report showed that a vaccine based on heat-killed pathogens induced spleen M-MDSCs that can be activated to kill dendritic cells (DCs), an additional mechanism that may help to explain the difficulties found to develop a very successful anti-pathogen vaccine (168). Tumor vaccines harness the tumor as the source of antigens and implement sequential immunomodulation to generate systemic and lasting antitumor immunity. Mechanism accounting for these is based on isolated patient-derived DCs, through pulsing them with tumor-associated antigens (TAAs) or neoantigens and maturation signals, followed by their reinfusion, or directly inject the antigens subcutaneously by activating DCs *in vivo* in patients (169). A major challenge facing the future of tumor vaccines for cancer treatments is to persist the cytotoxic T cell responses and overcome inhibitory signals from MDSCs in TME, understand the mechanisms of resistance to vaccines and to develop combination therapies that enhance antitumor immunity and durable responses. In a syngeneic B16F0 melanoma model and using tyrosinase related protein 1 (TRP1) as a vaccine antigen, it has been found that simultaneous delivery of IL-12 and a PD-L1-silencing shRNA was the only combination that exhibited therapeutically relevant anti-melanoma activities (170). Interestingly, the lentivector co-expressing IL-12 and the PD-L1 silencing shRNA was the only one that counteracted MDSC suppressive activities, potentially underlying the observed anti-melanoma therapeutic benefit (170). Prospective evaluation of candidate cancer treatment using ex vivo differentiated MDSCs highlights therapies with significant therapeutic potential and the therapy of the IL12-encoding combined with PD-L1 silencing lentivector vaccines demonstrated promising anti-melanoma activities. A prophylactic vaccine by employing exosomes derived from murine ESCs engineered to produce GM-CSF (ES-exo/GM-CSF vaccine) successfully protects mice from the outgrowth of an implantable form of murine lung cancer and provides protection against metastasized pulmonary tumors, by decreasing the frequencies of tumor infiltrating immunosuppressive immune cells, including Treg cells and MDSCs (171). Similar to this idea, treatment with sunitinib, which inhibits G-MDSCs prior to the vaccine composed of peptides of the tumor antigen survivin (SVX vaccine), could magnify the vaccine-mediated immune responses in a colorectal carcinoma mouse model (172). IFNa and 5-Aza-2’-deoxycytidine combined with a DC targeting DNA vaccine (a MIP3a fused vaccine targeting two common melanoma antigens, gp100 and trp2) exhibited greater tumor infiltration of DCs, and NK cells, as well as reduced levels of MDSCs in vaccinated groups in the B16F10 melanoma model (173). The combination therapy alters the tumor immune cell infiltration and elicits protective immune responses, but the underlying mechanisms needs to be explored. The advent of vaccines in multiple solid tumors has prompted the development of new therapeutic combinations that target MDSCs, modulating the TIME and the systemic antitumor response. Moreover, to explore new strategies to optimize the efficacy of standard immunotherapies, it is essential to find approaches that target MDSCs in antitumor immunization.

## 5 Concluding remarks

Tumor immunotherapy has undergone remarkable advances in recent years and has shown great potential for cancer patients. For most cancer patients, a favorable initial response to immunotherapy, is followed by limited responses and cancer relapse and recurrence, due to the multiple mechanisms inducing tumor immunosuppression (174). It also becomes more urgent and possible to reinforce the immune responses against tumors by ICB, adoptive cell transfer (ACT) therapy or tumor vaccines, and abolish TIME. Many studies found nonresponses to those therapies, mostly due to ignorance of the shape of the TIME after treatment. Thus, exploration of combination immunotherapeutic strategies coupled with other immunotherapy with reprogramming the TIME will be the top priority. It should be noted that MDSCs are known to suppress the anti-tumor immune response to induce host tolerance, support cancer stem cells and increase tumor angiogenesis and vascular maturation (175–178), suggesting that MDSC-targeted therapeutic approaches have broad implications in a wide range of cancer therapies in addition to immunotherapy. A growing number of studies have demonstrated that circulating MDSCs in cancer patients are a negative prognostic biomarker in predicting disease course, tumor stage, or metastatic spread (179). Thus, MDSCs have been recognized as a promising therapeutic target and prognostic biomarker for cancer patients, while the diversity, complexity, and heterogeneity of human MDSCs make it difficult to define their phenotypes accurately and uniformly in cancer. Therefore, it is necessary to investigate the phenotypes and characterizations of MDSCs in different types of tumors to establish the precise means to eliminate MDSCs. Although, the phenotypes and suppressive mechanisms seem to be shared among tumor MDSC subsets, it is necessary to identify and distinguish the differences in detail, in order to make more use of accurate individualized treatment regimens. We have reported here that numerous preclinical trails in mouse tumor models, have exhibited favorable efficacy by targeting MDSCs (61, 180).

The field of MDSC research still has more questions than answers. Better characterization of human MDSCs and a clearer understanding of whether MDSC-targeted cues will be of clinical significance are main priorities in this field. We do not yet know the outcomes of the ongoing clinical trials, including inhibition of MDSC immunosuppressive activity, blockade of MDSC recruitment and expansion, and promotion of MDSC differentiation into mature non-suppressive cells. However, reprogramming MDSCs in tumors, combined with newly developing immunotherapy, like ICB or ACT, seems to be a potential new approach to improve antitumor immunity, although adverse events of the treatment strategies should be taken into consideration. Removing the negative or suppressive immune response and improving the positive immune response is a theoretically ideal scheme to mediate anti-tumor immunity. However, the systemic depletion of suppressive cells, like Treg cells, also causes serious immune-related adverse events (181, 182). Hence, how to realize the immunoregulation in or around the tumor sites to augment antitumor immunity will be challenging. Therefore, developing therapeutic strategies targeting MDSC subpopulation is of paramount importance to improve the effectiveness of tumor therapy. We are at an interesting point in the translation of cancer immunotherapies where an improved knowledge of targeting MDSCs will be critical.

## Author contributions

All authors contributed to the article and approved the submitted version. XS and JD was responsible for the overall conceptualization, supervision and writing of the review. YZ was responsible for the draft of the original article, figures and tables.
